# Correction: Is the public sector of your country a diffusion borrower? Empirical evidence from Brazil

**DOI:** 10.1371/journal.pone.0191177

**Published:** 2018-01-09

**Authors:** Leno S. Rocha, Frederico S. A. Rocha, Thársis T. P. Souza

The files published for S1 and S2 Figs are incorrect. Please view the correct Supporting Information files below.

## Supporting information

S1 Fig Credit pleas per year along with the analytical solution provided by the logistic model. (TIF)

S2 Fig Number of pleaded credit operations per federated entity in Brazil from 2002 to 2015. Operations of municipalities aggregated at the state level. Considerable spatial hetegonenity in the number of loan pleas by federated entity can be observed.
(TIF)
