# Supplementary material for: Is the public sector of your country a diffusion borrower? Empirical evidence from Brazil
Source: PLoS One. 2017 Oct 5;12(10):e0185257. doi: 10.1371/journal.pone.0185257 (PMC5628819; doi:10.1371/journal.pone.0185257)
Supplement: S1 Appendix — (PDF) [file pone.0185257.s001.pdf]

Is the Public Sector of Your Country a Diffusion Borrower? Empirical Evidence from Brazil.

Leno S. Rocha[1*], Frederico S. A. Rocha[2], Thársis T. P. Souza[3]

1 Brazilian Treasury Secretariat, Ministry of Finance, Brasilia, DF, Brazil
2 Department of Computer Science, University of Utah, Salt Lake City, USA
3 Department of Computer Science, UCL, London, UK

* leno.rocha@tesouro.gov.br

S1 Appendix. Solution of the logistic diffusion process.

Recall Eq. 1, the differential equation of generalized growth, and consider it with $t \in \mathbb{R}^+$ and $n \in \mathbb{N}^*$:

$$\dot{N}(t) = \frac{dN(t)}{dt} = g(t)\{m^n - [N(t)]^n\}/n. \tag{4}$$

It is possible, using a notation abuse, to rewrite it as:

$$\frac{ndN(t)}{N(t)\{m^n - [N(t)]^n\}} = wdt \tag{5}$$

Making use of partial fractions, we have:

$$nm^{-n}\left\{\frac{1}{N(t)} + \frac{[N(t)]^{n-1}}{m^n - [N(t)]^n}\right\}dN(t) = wdt \tag{6}$$

By the chain rule, $d[N(t)]^n = n[N(t)]^{n-1}dN(t)$ and performing the integration, it follows:

$$n\ln|N(t)| - \ln|[N(t)]^n - m^n| = wm^n(t - t_0) \tag{7}$$

Once $m \geq N(t) \geq 0$, we can eliminate the modulus notation, and doing the exponentiation we have:

$$\frac{[N(t)]^n}{m^n - [N(t)]^n} = \frac{1}{m^n - 1}\exp[wm^n(t - t_0)] \tag{8}$$

in which t_0 is the initial moment, that for methodological definition is the first day of the year, i.e, $t_0 = 1$. Finally, isolating the function of interest we have:

$$N(t) = m\{1 + (m^n - 1)\exp[-wm^n(t - 1)]\}^{\frac{-1}{n}} \tag{9}$$

Assuming $n = 1$, which makes the growth expressed in the differential Equation 1 to be proportional to an unidimensional distance, one arrives at the formula of Equation 2.

References

1. Matesanz D, Ortega GJ. Sovereign public debt crisis in Europe. A network analysis. Physica A: Statistical Mechanics and its Applications. 2015;436:756 – 766. doi:http://dx.doi.org/10.1016/j.physa.2015.05.052.

2. Panizza U, Presbitero AF. Public debt and economic growth: Is there a causal effect? Journal of Macroeconomics. 2014;41:21 – 41. doi:http://dx.doi.org/10.1016/j.jmacro.2014.03.009.

3. Bua G, Pradelli J, Presbitero AF. Domestic public debt in Low-Income Countries: Trends and structure. Review of Development Finance. 2014;4(1):1 – 19. doi:http://dx.doi.org/10.1016/j.rdf.2014.02.002.

4. Carranza L, Daude C, Melguizo A. Public infrastructure investment and fiscal sustainability in Latin America: incompatible goals? Journal of Economic Studies. 2014;41(1):29–50. doi:http://dx.doi.org/10.1108/JES-03-2012-0036.

5. IMF guidelines for public debt management; 2014. http://bit.ly/231Id9s.

6. Spilioti S, Vamvoukas G. The impact of government debt on economic growth: An empirical investigation of the Greek market. The Journal of Economic Asymmetries. 2015;12(1):34 – 40. doi:http://dx.doi.org/10.1016/j.jeca.2014.10.001.

7. Mitze T, Matz F. Public debt and growth in German federal states: What can Europe learn? Journal of Policy Modeling. 2015;37(2):208 – 228. doi:http://dx.doi.org/10.1016/j.jpolmod.2015.02.003.

8. Jenkner E, Lu Z. Subnational credit risk and sovereign bailouts – Who pays the premium? IMF working paper WP14/20. 2014;.

9. Buiatti C, Carmeci G, Mauro L. The origins of the public debt of Italy: Geographically dispersed interests? Journal of Policy Modeling. 2014;36(1):43–62.

10. Shone R. Economic Dynamics: Phase diagrams and their economic application. Cambridge University Press; 2002.

11. Li L. Patch invasion in a spatial epidemic model. Applied Mathematics and Computation 258, 342–349 (2015). http://dx.doi.org/10.1016/j.amc.2015.02.006.

12. Tsoularis, A. and Wallace, J. (2002). Analysis of logistic growth models. *Mathematical Biosciences*, 179(1):21–55.

13. Sun, G.Q., Jusup, M., Jin, Z., Wang, Y., Wang, Z.: Review. Physics of Life Reviews 19(Complete), 43–73 (2016)

14. Sun, G.Q., Chakraborty, A., Liu, Q.X., Jin, Z., Anderson, K.E., Li, B.L.: Influence of time delay and nonlinear diffusion on herbivore outbreak. Communications in Nonlinear Science and Numerical Simulation 19(5), 1507 – 1518 (2014), http://www.sciencedirect.com/science/article/pii/S1007570413004164

15. Gompertz B. On the nature of the function expressive of the law of human mortality, and on a new mode of determining the value of life contingencies. Philosophical transactions of the Royal Society of London. 1825; p. 513–583.

16. Brazilian Federal Senate, Resolution 43; 2001. http://bit.ly/1Ud7xJg.

17. Constitution of the Federative Republic of Brazil; 1988. http://bit.ly/1GLK9tA.

18. Brazilian National Monetary Council, Resolution nº 2.827; 2001. http://bit.ly/23WyNl7.

19. Brazilian Presidency, Decree nº 3.502; 2000. http://bit.ly/1QxvJPu.

20. SADIPEM. Brazilian National Treasury Secretariat: Historical data; 2015. https://sadipem.tesouro.gov.br/.

21. Sakurai SN, Menezes-Filho N. Opportunistic and partisan election cycles in Brazil: new evidence at the municipal level. Public Choice. 2010;148(1):233–247. doi:10.1007/s11127-010-9654-1.

22. Alesina A, Cohen GD, Roubini N. Macroeconomic Policy and Elections in OECD Democracies. National Bureau of Economic Research; 1991. 3830. Available from: http://www.nber.org/papers/w3830.

23. United Kingdom Government, Department of Communities and Local Government; 2015. http://bit.ly/21deqLE.

24. Japan Finance Organization for Municipalities; 2016. http://www.jfm.go.jp/en/about/financing.html.

25. Ministry of Internal Affairs and Communications, Local Government Bond System and Market in Japan; 2016. http://www.jlgc.org.uk/en/pdfs/MIC%20LGB.pdf.
