# Supplementary material for: Is the public sector of your country a diffusion borrower? Empirical evidence from Brazil
Source: PLoS One. 2017 Oct 5;12(10):e0185257. doi: 10.1371/journal.pone.0185257 (PMC5628819; doi:10.1371/journal.pone.0185257)
Supplement: S2 Appendix — (PDF) [file pone.0185257.s002.pdf]

Is the Public Sector of Your Country a Diffusion Borrower? Empirical Evidence from Brazil.

Leno S. Rocha[1*], Frederico S. A. Rocha[2], Thársis T. P. Souza[3]

1 Brazilian Treasury Secretariat, Ministry of Finance, Brasilia, DF, Brazil
2 Department of Computer Science, University of Utah, Salt Lake City, USA
3 Department of Computer Science, UCL, London, UK

* leno.rocha@tesouro.gov.br

S2 Appendix. Derivation of Gompertz equation.

Writing powers via logarithms and making $n \to 0$, which corresponds to a growth proportional to a logarithmic distance [15] in the differential Equation 1, we have:

$$\lim_{n\to 0} \dot{N}(t) = wN(t) \lim_{n\to 0} \frac{\exp(n \ln m) - \exp[n \ln N(t)]}{n} \tag{10}$$

Resorting to power series of the Napier's exponential function, it follows:

$$\dot{N}(t) = wN(t) \lim_{n\to 0} \frac{1}{n} \left\{ \sum_{i=0}^{\infty} \frac{(n \ln m)^i}{i!} - \sum_{i=0}^{\infty} \frac{[n \ln N(t)]^i}{i!} \right\} \tag{11}$$

Sorting out the two first elements of each summation, we have:

$$\dot{N}(t) = wN(t) \left\{ \ln \left[\frac{m}{N(t)} \right] + \lim_{n\to 0} \sum_{i=2}^{\infty} n^{i-1} \{ (\ln m)^i / i! - [\ln N(t)]^i / i! \} \right\} \tag{12}$$

The limit of the remaining summation tends to zero, so that we have, as already stated, a logarithmic distance:

$$\dot{N}(t) = wN(t) \ln \left[\frac{m}{N(t)} \right]. \tag{13}$$

Rewriting the Equation 13, again with notation abuse, follows:

$$\frac{d\{\ln[N(t)/m]\}}{\ln[N(t)/m]} = -wdt. \tag{14}$$

Integrating the Equation 14 we have:

$$\ln |\ln[N(t)/m]| - \ln |\ln m| = -w(t-1). \tag{15}$$

Performing two exponentiations, we arrive at the Gompertz Equation:

$$\left| \frac{\ln[N(t)/m]}{\ln m} \right| = \exp[-w(t-1)] \tag{16}$$

$$N(t) = m \exp\{-(\ln m) exp[-w(t-1)]\} \tag{17}$$

In the original Gompertz function, $b = -$ (ln m) $exp(w)$ and $c = w$, as can be seen in Equation 17 . But as previously mentioned, we allowed the parameters to be independent in order to improve the adherence to the data. Adopting this relaxation one arrives at the Equation 3.

References

1. Matesanz D, Ortega GJ. Sovereign public debt crisis in Europe. A network analysis. Physica A: Statistical Mechanics and its Applications. 2015;436:756 – 766. doi:http://dx.doi.org/10.1016/j.physa.2015.05.052.

2. Panizza U, Presbitero AF. Public debt and economic growth: Is there a causal effect? Journal of Macroeconomics. 2014;41:21 – 41. doi:http://dx.doi.org/10.1016/j.jmacro.2014.03.009.

3. Bua G, Pradelli J, Presbitero AF. Domestic public debt in Low-Income Countries: Trends and structure. Review of Development Finance. 2014;4(1):1 – 19. doi:http://dx.doi.org/10.1016/j.rdf.2014.02.002.

4. Carranza L, Daude C, Melguizo A. Public infrastructure investment and fiscal sustainability in Latin America: incompatible goals? Journal of Economic Studies. 2014;41(1):29–50. doi:http://dx.doi.org/10.1108/JES-03-2012-0036.

5. IMF guidelines for public debt management; 2014. `http://bit.ly/231Id9s`.

6. Spilioti S, Vamvoukas G. The impact of government debt on economic growth: An empirical investigation of the Greek market. The Journal of Economic Asymmetries. 2015;12(1):34 – 40. doi:http://dx.doi.org/10.1016/j.jeca.2014.10.001.

7. Mitze T, Matz F. Public debt and growth in German federal states: What can Europe learn? Journal of Policy Modeling. 2015;37(2):208 – 228. doi:http://dx.doi.org/10.1016/j.jpolmod.2015.02.003.

8. Jenkner E, Lu Z. Subnational credit risk and sovereign bailouts – Who pays the premium? IMF working paper WP14/20. 2014;.

9. Buiatti C, Carmeci G, Mauro L. The origins of the public debt of Italy: Geographically dispersed interests? Journal of Policy Modeling. 2014;36(1):43–62.

10. Shone R. Economic Dynamics: Phase diagrams and their economic application. Cambridge University Press; 2002.

11. Li L. Patch invasion in a spatial epidemic model. Applied Mathematics and Computation 258, 342–349 (2015). `http://dx.doi.org/10.1016/j.amc.2015.02.006`.

12. Tsoularis, A. and Wallace, J. (2002). Analysis of logistic growth models. *Mathematical Biosciences*, 179(1):21–55.

13. Sun, G.Q., Jusup, M., Jin, Z., Wang, Y., Wang, Z.: Review. Physics of Life Reviews 19(Complete), 43–73 (2016)

14. Sun, G.Q., Chakraborty, A., Liu, Q.X., Jin, Z., Anderson, K.E., Li, B.L.: Influence of time delay and nonlinear diffusion on herbivore outbreak. Communications in Nonlinear Science and Numerical Simulation 19(5), 1507 – 1518 (2014), `http://www.sciencedirect.com/science/article/pii/S1007570413004164`

15. Gompertz B. On the nature of the function expressive of the law of human mortality, and on a new mode of determining the value of life contingencies. Philosophical transactions of the Royal Society of London. 1825; p. 513–583.

16. Brazilian Federal Senate, Resolution 43; 2001. http://bit.ly/1Ud7xJg.

17. Constitution of the Federative Republic of Brazil; 1988. http://bit.ly/1GLK9tA.

18. Brazilian National Monetary Council, Resolution nº 2.827; 2001. http://bit.ly/23WyNl7.

19. Brazilian Presidency, Decree nº 3.502; 2000. http://bit.ly/1QxvJPu.

20. SADIPEM. Brazilian National Treasury Secretariat: Historical data; 2015. https://sadipem.tesouro.gov.br/.

21. Sakurai SN, Menezes-Filho N. Opportunistic and partisan election cycles in Brazil: new evidence at the municipal level. Public Choice. 2010;148(1):233–247. doi:10.1007/s11127-010-9654-1.

22. Alesina A, Cohen GD, Roubini N. Macroeconomic Policy and Elections in OECD Democracies. National Bureau of Economic Research; 1991. 3830. Available from: http://www.nber.org/papers/w3830.

23. United Kingdom Government, Department of Communities and Local Government; 2015. http://bit.ly/21deqLE.

24. Japan Finance Organization for Municipalities; 2016. http://www.jfm.go.jp/en/about/financing.html.

25. Ministry of Internal Affairs and Communications, Local Government Bond System and Market in Japan; 2016. http://www.jlgc.org.uk/en/pdfs/MIC%20LGB.pdf.
