# Supplementary material for: Is the public sector of your country a diffusion borrower? Empirical evidence from Brazil
Source: PLoS One. 2017 Oct 5;12(10):e0185257. doi: 10.1371/journal.pone.0185257 (PMC5628819; doi:10.1371/journal.pone.0185257)
Supplement: S3 Appendix — (PDF) [file pone.0185257.s003.pdf]

Is the Public Sector of Your Country a Diffusion Borrower? Empirical Evidence from Brazil.

Leno S. Rocha[1*], Frederico S. A. Rocha[2], Thársis T. P. Souza[3]

1 Brazilian Treasury Secretariat, Ministry of Finance, Brasilia, DF, Brazil
2 Department of Computer Science, University of Utah, Salt Lake City, USA
3 Department of Computer Science, UCL, London, UK

* leno.rocha@tesouro.gov.br

S3 Appendix. Considerations about spatial effects.
The incorporation of space explicitly into mathematical models may rise more understanding about dynamic systems. In the epidemiological area, it has shed light to new knowledge such as pathogen extinction even with the basic reproduction number greater than unity, the eventual occurrence of chaos, spatio-temporal patterns and various emergent properties, with results prone to be transcending the bounds of several research areas [13].

With regards to ecosystems dynamics, spatial considerations showed, for example, other mechanism for outbreak of herbivore [14]. In the case of credit operations of federated entities in Brazil, object of study of the present article, although the model represented by Eq. 3 is parsimonious and led to good results, the inclusion of spatial dynamics may provide some insights, particularly because, as already discussed, there is considerable heterogeneity in the number of credit operation pleas by federated entity.

This heterogeneity might be interpreted from the spatial point of view, as suggests S2 Fig which shows the concentration of the number of operations already pleaded, from 2002 to 2015, according to the locality of the federated entity. The distribution of pleas indicates "hot areas" of loans requests from the southeast towards the south of Brazil.

Consider a coordinate system $(x, y) \in \mathbb{R}^2$, established to refer to the Brazilian territory, the time variable $t \in \mathbb{R}^+$ and the parameter $n \in \mathbb{N}^*$. Departing from Eq. 1, one can introduce the spatial dynamics to obtain the partial differential equation of generalized growth as follow:

$$\frac{\partial N(x,y,t)}{\partial t} = g(t)\{m^n - [N(t)]^n\}/n + D\nabla^2 N(x,y,t) \tag{1}$$

wherein D is a spatial diffusion coefficient [11] and $\nabla^2 = \partial^2/\partial x^2 + \partial^2/\partial y^2$ is the usual Laplacian operator. We intend to explore this model in the future, especially in order to verify the occurrence of spatial patterns, the underlying mechanisms and related emergent properties.

References

1. Matesanz D, Ortega GJ. Sovereign public debt crisis in Europe. A network analysis. Physica A: Statistical Mechanics and its Applications. 2015;436:756 – 766. doi:http://dx.doi.org/10.1016/j.physa.2015.05.052.

2. Panizza U, Presbitero AF. Public debt and economic growth: Is there a causal effect? Journal of Macroeconomics. 2014;41:21 – 41. doi:http://dx.doi.org/10.1016/j.jmacro.2014.03.009.

3. Bua G, Pradelli J, Presbitero AF. Domestic public debt in Low-Income Countries: Trends and structure. Review of Development Finance. 2014;4(1):1 – 19. doi:http://dx.doi.org/10.1016/j.rdf.2014.02.002.

4. Carranza L, Daude C, Melguizo A. Public infrastructure investment and fiscal sustainability in Latin America: incompatible goals? Journal of Economic Studies. 2014;41(1):29–50. doi:http://dx.doi.org/10.1108/JES-03-2012-0036.

5. IMF guidelines for public debt management; 2014. `http://bit.ly/231Id9s`.

6. Spilioti S, Vamvoukas G. The impact of government debt on economic growth: An empirical investigation of the Greek market. The Journal of Economic Asymmetries. 2015;12(1):34 – 40. doi:http://dx.doi.org/10.1016/j.jeca.2014.10.001.

7. Mitze T, Matz F. Public debt and growth in German federal states: What can Europe learn? Journal of Policy Modeling. 2015;37(2):208 – 228. doi:http://dx.doi.org/10.1016/j.jpolmod.2015.02.003.

8. Jenkner E, Lu Z. Subnational credit risk and sovereign bailouts – Who pays the premium? IMF working paper WP14/20. 2014;.

9. Buiatti C, Carmeci G, Mauro L. The origins of the public debt of Italy: Geographically dispersed interests? Journal of Policy Modeling. 2014;36(1):43–62.

10. Shone R. Economic Dynamics: Phase diagrams and their economic application. Cambridge University Press; 2002.

11. Li L. Patch invasion in a spatial epidemic model. Applied Mathematics and Computation 258, 342–349 (2015). `http://dx.doi.org/10.1016/j.amc.2015.02.006`.

12. Tsoularis, A. and Wallace, J. (2002). Analysis of logistic growth models. *Mathematical Biosciences*, 179(1):21–55.

13. Sun, G.Q., Jusup, M., Jin, Z., Wang, Y., Wang, Z.: Review. Physics of Life Reviews 19(Complete), 43–73 (2016)

14. Sun, G.Q., Chakraborty, A., Liu, Q.X., Jin, Z., Anderson, K.E., Li, B.L.: Influence of time delay and nonlinear diffusion on herbivore outbreak. Communications in Nonlinear Science and Numerical Simulation 19(5), 1507 – 1518 (2014), `http://www.sciencedirect.com/science/article/pii/S1007570413004164`

15. Gompertz B. On the nature of the function expressive of the law of human mortality, and on a new mode of determining the value of life contingencies. Philosophical transactions of the Royal Society of London. 1825; p. 513–583.

16. Brazilian Federal Senate, Resolution 43; 2001. `http://bit.ly/1Ud7xJg`.

17. Constitution of the Federative Republic of Brazil; 1988. `http://bit.ly/1GLK9tA`.

18. Brazilian National Monetary Council, Resolution nº 2.827; 2001. `http://bit.ly/23WyNl7`.

19. Brazilian Presidency, Decree nº 3.502; 2000. `http://bit.ly/1QxvJPu`.

20. SADIPEM. Brazilian National Treasury Secretariat: Historical data; 2015. https://sadipem.tesouro.gov.br/.

21. Sakurai SN, Menezes-Filho N. Opportunistic and partisan election cycles in Brazil: new evidence at the municipal level. Public Choice. 2010;148(1):233–247. doi:10.1007/s11127-010-9654-1.

22. Alesina A, Cohen GD, Roubini N. Macroeconomic Policy and Elections in OECD Democracies. National Bureau of Economic Research; 1991. 3830. Available from: http://www.nber.org/papers/w3830.

23. United Kingdom Government, Department of Communities and Local Government; 2015. http://bit.ly/21deqLE.

24. Japan Finance Organization for Municipalities; 2016. http://www.jfm.go.jp/en/about/financing.html.

25. Ministry of Internal Affairs and Communications, Local Government Bond System and Market in Japan; 2016. http://www.jlgc.org.uk/en/pdfs/MIC%20LGB.pdf.
