# Supplementary material for: Is the public sector of your country a diffusion borrower? Empirical evidence from Brazil
Source: PLoS One. 2017 Oct 5;12(10):e0185257. doi: 10.1371/journal.pone.0185257 (PMC5628819; doi:10.1371/journal.pone.0185257)
Supplement: S2 Fig — Operations of municipalities aggregated at the state level. Considerable spatial hetegonenity in the number of loan pleas by federated entity can be observed. (PDF) [file pone.0185257.s005.pdf]

Is the Public Sector of Your Country a Diffusion Borrower? Empirical Evidence from Brazil.

Leno S. Rocha[1*], Frederico S. A. Rocha[2], Thársis T. P. Souza[3]

1 Brazilian Treasury Secretariat, Ministry of Finance, Brasilia, DF, Brazil
2 Department of Computer Science, University of Utah, Salt Lake City, USA
3 Department of Computer Science, UCL, London, UK

* leno.rocha@tesouro.gov.br

S2 Fig. Number of pleaded credit operations per federated entity in Brazil from 2002 to 2015. Operations of municipalities aggregated at the state level. Considerable spatial heterogeneity in the number of loan pleas by federated entity can be observed.
