# Supplementary figures and images for: Correction: Is the public sector of your country a diffusion borrower? Empirical evidence from Brazil
Source: PLoS One. 2018 Jan 9;13(1):e0191177. doi: 10.1371/journal.pone.0191177 (PMC5760080; doi:10.1371/journal.pone.0191177)

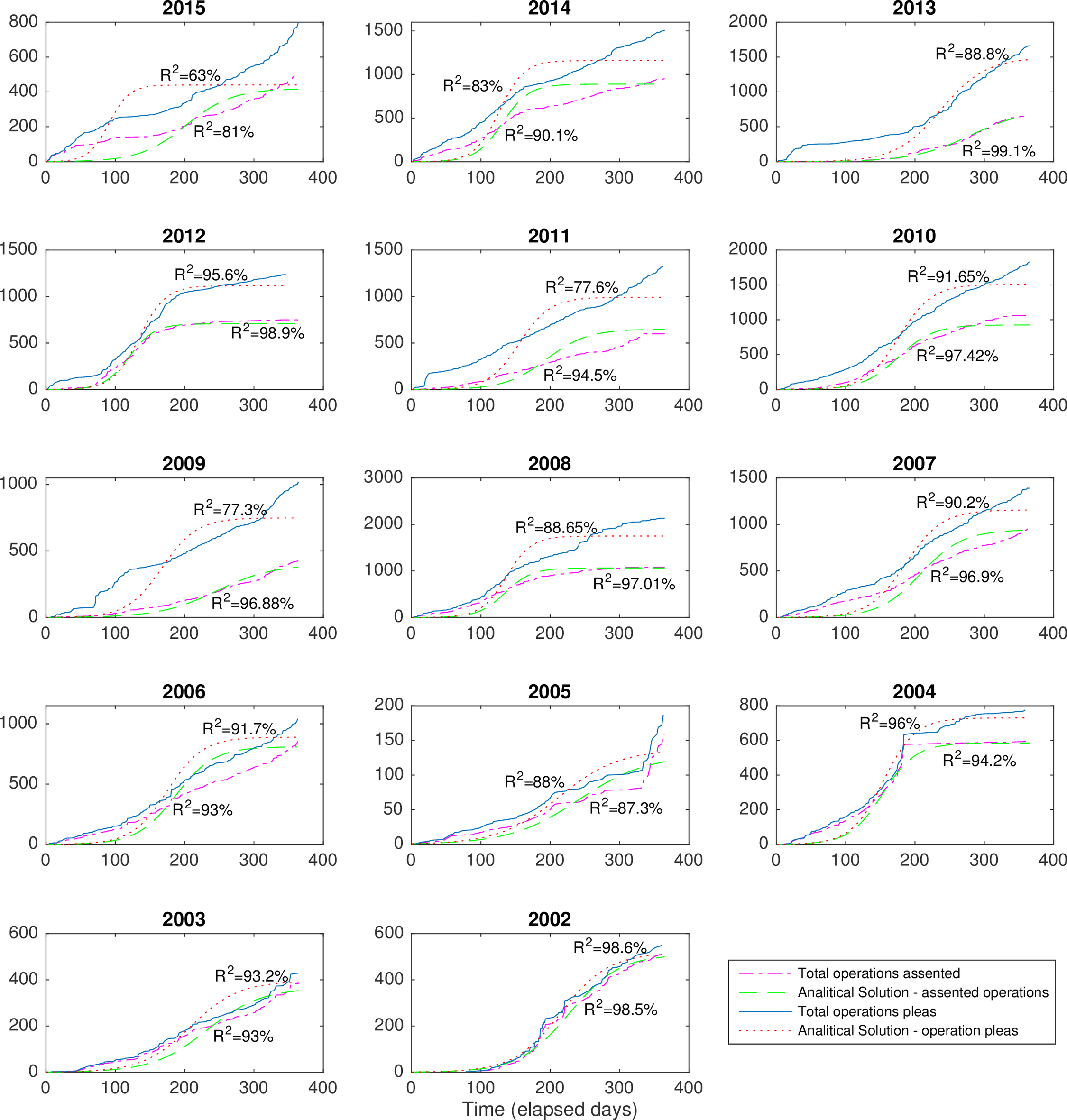

Supplement: S1 Fig — (TIF) [file pone.0191177.s001.tif]

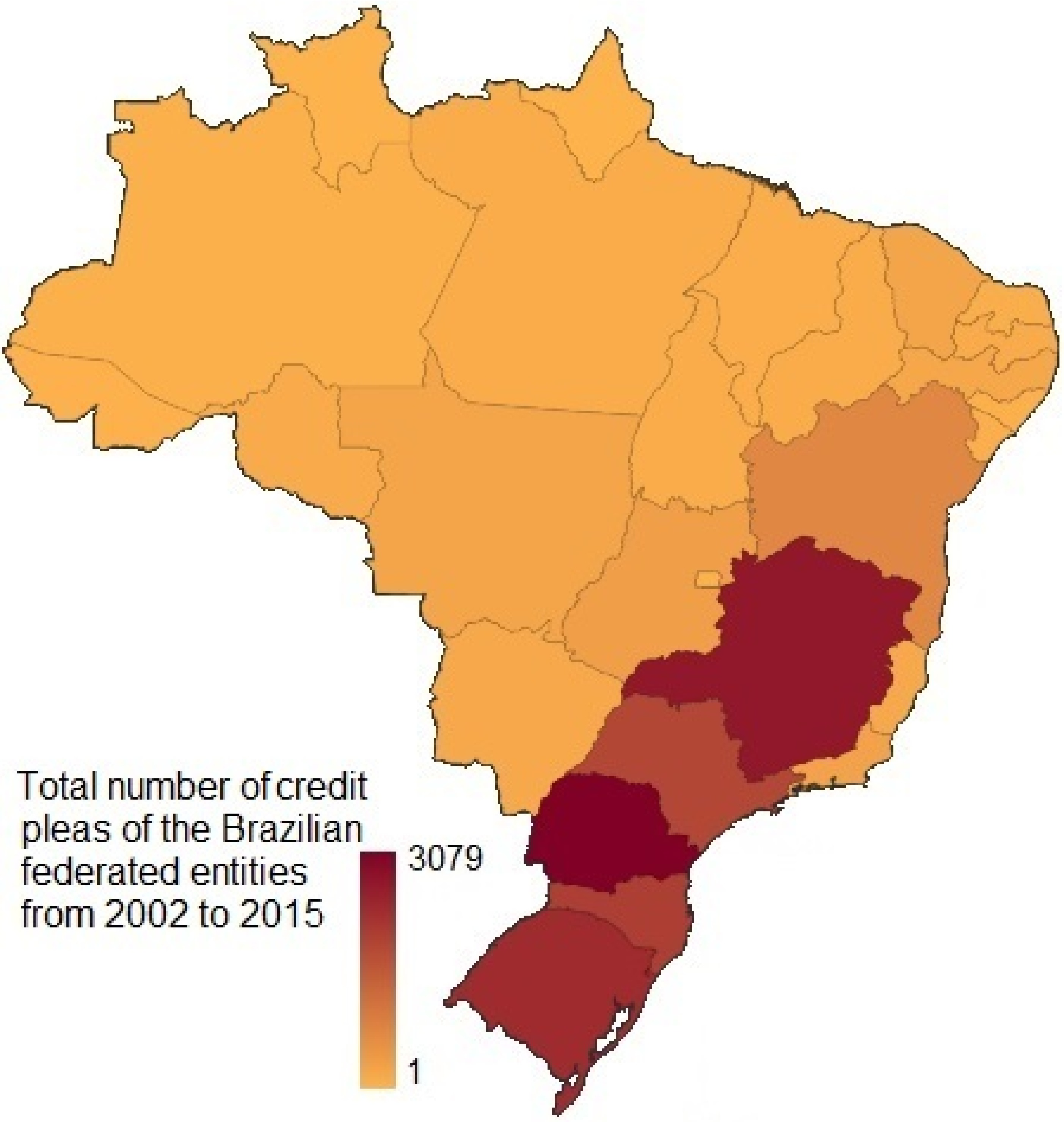

Supplement: S2 Fig — Operations of municipalities aggregated at the state level. Considerable spatial hetegonenity in the number of loan pleas by federated entity can be observed. (TIF) [file pone.0191177.s002.tif]
